# Rapid Detection to Differentiate Hypervirulent *Klebsiella pneumoniae* (hvKp) From Classical *K. pneumoniae* by Identifying *peg-344* With Loop-Mediated Isothermal Amplication (LAMP)

**DOI:** 10.3389/fmicb.2020.01189

**Published:** 2020-06-04

**Authors:** Wenjian Liao, Dan Long, Qisen Huang, Dandan Wei, Xiaobing Liu, Lagen Wan, Yuling Feng, Wei Zhang, Yang Liu

**Affiliations:** ^1^Department of Respiratory Medicine, The First Affiliated Hospital of Nanchang University, Nanchang, China; ^2^Department of Clinical Microbiology, The First Affiliated Hospital of Nanchang University, Nanchang, China; ^3^Department of Endocrinology Medicine, The Third Hospital of Nanchang, Nanchang, China; ^4^Medicine College, Nanchang University, Nanchang, China

**Keywords:** hypervirulent *K. pneumoniae*, loop-mediated isothermal amplication, *peg-344*, rapid, easy

## Abstract

**Objectives:**

To establish a rapid molecular diagnostics of hvKp using the *peg-344* loop-mediated isothermal amplification technique (LAMP).

**Methods:**

In all, 28 *K. pneumoniae* strains isolated from the blood of patients were used for the *peg-344* LAMP. *K. pneumoniae* NTUH-K2044 and *K. pneumoniae* ATCC700603 were used as positive control and negative control, respectively. For comparison, all the results were detected in a polymerase chain reaction (PCR), which was considered the gold standard for the detection of the gene. Mouse lethality assay, and Serum killing assay were also used to determine the virulence phenotype of *K. pneumoniae*.

**Results:**

We determined the specificity and sensitivity of the primers for *peg-344* detection in the LAMP reactions. This LAMP assay was able to specifically differentiate hvKp from classical *K. pneumoniae* (cKp) at 65°C, which was 100-fold more sensitive than a PCR assay for *peg-344* detection. The virulence phenotype of *K. pneumoniae* detected by LAMP was as precise as by Mouse lethality assay and Serum killing assay.

**Conclusion:**

The LAMP assay is easy to perform and rapid. Therefore, it can be routinely applied to differentiate hvKp from cKp in the clinical laboratory.

## Introduction

A new variant of *Klebsiella pneumoniae*, designated as hypervirulent *K. pneumoniae* (hvKp), was first described to cause a clinical syndrome of community-acquired *K. pneumoniae* infections in 1986 in Taiwan (Li et al., 2014). hvKp is an evolving pathotype that is considered to be more virulent than classical *K. pneumoniae* (cKp) both in clinical research and basic research. hvKp infection is highly characterized by its ability to infect healthy individuals of any age and the tendency of infected patients to have multiple infection sites and/or subsequent metastatic spread (Shon et al., 2013). Traditional methods for detecting hvKp include Colony morphology, String test, Serum killing assay, Mouse lethality assay, Galleria mellonella infection model, etc. (Shi et al., 2018; Liu et al., 2019). These methods are too time-consuming to assess virulence. Recently, it has been shown that several biomarkers and quantitative siderophore production can accurately predict hvKp strains, which could lead to the development of diagnostic tests for the clinical laboratory for optimal patient care as well as for epidemiological surveillance and research (Russo and Marr, 2019). The presence of biomarkers on virulence plasmids has been shown to most accurately distinguish hvKp from cKp strains. Fortunately, a previous study found *peg-344*, *iroB*, *iucA*, *prmpA*, *prmpA2*, and siderophore production greater than 30 μg/mL had been shown to accurately differentiate hvKp from cKp strains. Furthermore, the gene *peg-344* had the highest accuracy, sensitivity, and specificity according to the odds ratio in the performance characteristics of the trait assessed as markers to identify hvKp (Russo et al., 2018). Although the function of PEG344 is unclear, homology modeling suggests that it may be a transporter located on the inner membrane. By measuring survival and competition experiments, PEG344 is completely necessary for maximal virulence in a pneumonia model, but does not seem to promote systemic infection occurs after a subcutaneous attack challenge (Bulger et al., 2017).

Loop-mediated isothermal amplification (LAMP) is a new technology for nucleic acid-specific amplification (Notomi et al., 2000). Overcoming some of the drawbacks and limitations of PCR, LAMP has been widely used in the diagnostic testing of infectious agents. In many studies, the sensitivity of LAMP to detect target sequences had shown to be 10 times higher than PCR (Jeong et al., 2013; Dong et al., 2015; Kitamura et al., 2017). The reaction time of LAMP is shorter than that of PCR, because LAMP is performed at isothermal temperature (60–65°C), and amplification products can be observed without electrophoresis. In addition, because LAMP does not require a thermal cycler, it is cheaper than other molecular diagnostic methods (Bakhtiari et al., 2016). Therefore, by detecting the presence of the virulence gene *peg-344*, the purpose of this study was to establish the rapid molecular diagnostics of hvKp using LAMP technology since it is low cost and easy to perform.

## Materials and Methods

### Bacterial Isolates

To evaluate the specificity of the LAMP assay for detecting *peg-344*, a total of 28 *K. pneumoniae* isolates collected from the blood of patients were selected. Different kinds of standard strains were also included in this study to evaluate the specificity of hvKp (Table 1). The *K. pneumoniae* NTUH-K2044 was used as the positive control.

**TABLE 1:** Different kinds of standard strains used to LAMP evaluation and the conditions of PCR reactions.

**Bacterial species evaluated**	**PCR**	**LAMP**
*Klebsiella pneumoniae* NTUH-K2044	+	+
Classic *Klebsiella pneumoniae* ATCC700603	−	−
*Vibrio parahaemolyticus* AP125	−	−
*Escherichia coli* DP13	−	−
*Streptococcus viridans* DZ17	−	−
*Staphylococcus aureus* AP108	−	−
*Enterococcus tyrazoides* DZ208	−	−
*Enterococcus faecalis* DP119	−	−
*Stenotrophomonas maltophilia* CZ6	−	−
*Enterobacter cloacae* DZ134	−	−
*Candida albicans* OP245	−	−
*Candida cress* OP206	−	−
*Pseudomonas aeruginosa* CZ5	−	−
*Enterobacter aerogenes* OP205	−	−
*Campylobacter jejunii* CZ22	−	−
*Serratia marcescens* CZ7	−	−

### LAMP Assay

The Kit Bst 2.0 DNA Polymerase (Eiken Chemical Co. Ltd., Tokyo, Japan) was used for the LAMP reaction in a volume of 25 μL. The reaction mixture was composed of 3 primers, viz., a pair of inner primers (FIP and BIP, 80pmol of each), a pair of outer primers (F3 and B3, 5pmol of each), a pair of loop primers (LB and LF, 20 pmol of each), which would speed up the LAMP reaction, 1 μL of Bst DNA polymerase (8 units), 2 μL of DNA template, and 12.5 μL of the reaction mix available in the kit. The LAMP reaction was carried out under isothermal conditions, at 65°C, for 60 min and then stopped at 85°C for 5 min (Vergara et al., 2020).

### Primer Design

We designed the candidate LAMP primer sets from the nucleotide sequence of *peg-344* (GenBank accession number BAH65947.1) using Primer Explorer V4 software^1^ (Nakano et al., 2015; Table 2).

**TABLE 2:** LAMP primers used in this study.

**Assay**	**Primer**	**Sequence (5′–3′)**
	F3	TGGGGTTATTCTTTCGCT
	B3	TTTCCAAGCTTACTGCAATT
	FIP	CCAGCAAAACAGCCTAAATACATTGTGGGGA
		GTATCTTTGAGAGG
	BIP	TTGGGATACTGTGCTATTTTTCTCTGGGAAGA
		TGAGAAATACGAGC
	LF	CGCCTCCGTGATGAGGATG
	LB	GCAGAAAAGGGCTAGCGC

### DNA Extraction

Genomic DNA was extracted by boiling bacterial colonies in sterile distilled water for 10 min. After centrifugation, we use the supernatant as a DNA template (Wei et al., 2015).

### PCR Assay

PCR amplification was performed with the outer primer (F3 and B3) as described previously (Liao et al., 2019). The PCR conditions for *peg-344* was: 95°C for 5 min, followed by 30 repeated cycles of 30 s at 95°C, 30 s of annealing at 56°C and 1 min of extension at 72°C, followed by 7 min as a final extension at 72°C. hvKP marker genes (*iroB*, *iucA*, *rmpA*, *rmpA2*) were also identified using PCR amplification as described previously (Russo et al., 2018). All the PCR products were purified and sequenced and the sequences were compared with the reference deposited in the GenBank nucleotide database.

### Determination of the Sensitivity of LAMP Assay

**FIGURE 1:**
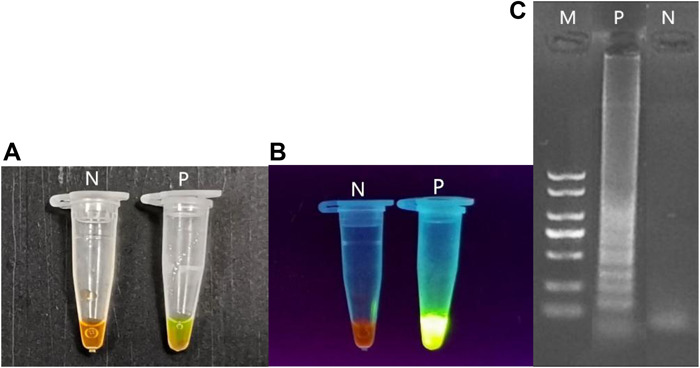
Interpretation of LAMP amplification results.LAMP amplification results: P: Positive control, N: Negative control. **(A)** Under natural light, after adding SYBR green I P: yellow-green, N: Dark yellow. **(B)** Under UV light, after adding SYBR green I, P: bright green, N: Dark orange. **(C)** After being electrophoresed on a 2% agarose gel, P: Stepped band, N: None.

To determine the sensitivity of the LAMP assay for detecting *peg-344*, serial dilutions of DNA from cultivated *K. pneumoniae* NTUH-K2044 cells were used. The products amplified by LAMP and PCR were electrophoresed on a 2% agarose gel, stained with ethidium bromide, and observed under a UV lamp. The LAMP products were also added into SYBR green I to observe the color change under nature and UV light. The interpretation of LAMP amplification results was shown in Figure 1. To confirm the accuracy of the LAMP reaction, we purified and sequenced the LAMP products, and then we analyzed the sequences with the BLAST program at the NCBI homepage^2^.

### Serum Killing Assay

Serum killing assay was performed as described previously (Liu et al., 2019). Briefly, prior to the assay, serum separated from 10 healthy individuals’ blood was stored at −80°C. A 10^6^ CFU of bacteria-containing inoculum prepared from the mid-log phase was reacted with 75% pooled human sera. The final mixture was incubated at 37°C, and we obtained viable counts at 0, 1, and 3 h, respectively. The response to serum killing in terms of viable counts was scored using six grades classified as serum sensitive (grade 1 or 2), intermediately sensitive (grade 3 or 4) or serum resistant (grade 5 or 6).

### Mouse Lethality Assay

Determination of the virulence of KP in mouse lethality tests and the medium lethal dose (LD50, expressed as colony-forming units) was performed as previously described (Yu et al., 2008). In brief, a graded dose of 10^1^–10^6^ CFU of each strain in 10-fold serial dilutions in 0.1 mL of normal saline was injected intraperitoneally into mice (4 mice for each dose of inoculum). All inoculated mice were recorded daily for survival. Interpretation of virulence was referred to reference (Siu et al., 2012).

### Ethics Statement

The study has been evaluated by the Ethics Committee of the First Affiliated Hospital of Nanchang University. Patients involved in the study were anonymized, no informed consent was acquired because of the retrospective study.

## Results

### The Most Appropriate Temperature for *peg-344* LAMP

By incubating the reaction mixture at 60–69°C, the optimum temperature was determined based on the LAMP amplification efficiency. Turbidity measurements showed that the optimal temperature for the LAMP amplification was 65°C. The turbidity was also monitored to observe the efficiency of the LAMP reaction in the absence of a loop primer at 65°C, and we found that the start-up of the amplification took about 20 or 40 min, with or without a loop primer, respectively. Thus, compared with amplification without loop primers, the use of this primer can reduce the time required for amplification by 50%.

### Specificity of *peg-344* LAMP

**FIGURE 2:**
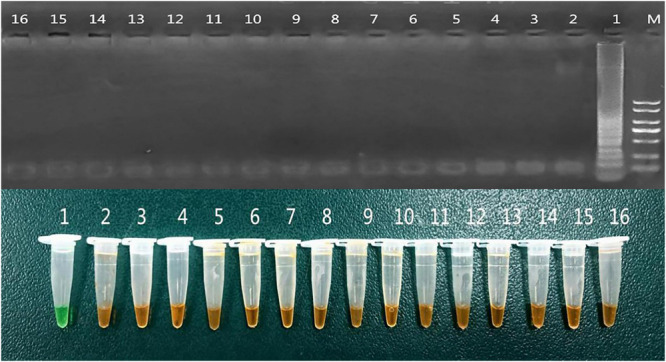
Specificity of *peg-344* LAMP. The specificity of the LAMP assay for detecting *peg-344* producers by assessing its reactivity with other kinds of standard strains. 1: *Klebsiella pneumoniae* NTUH-K2044 2-16: as shown in Table 1.

The specificity of the LAMP test to detect *peg-344* producers was evaluated by assessing its reactivity with other types of standard strains. As shown in Table 1 and Figure 2, We observed that DNA could be amplified from hvKp (*K. pneumoniae* NTUH-K2044) specifically by LAMP, but could not be amplified from other standards strains or the negative control (distilled water). We confirmed the accuracy of LAMP amplification by sequencing the amplified products with internal primers. The obtained sequence was the same as the expected nucleotide sequence of *peg-344*. Thus, this assay was highly specific for the detection of *peg-344* from hvKp. We also verified the accuracy of LAMP amplification by PCR amplification in the clinical *K. pneumoniae* strains (as shown in Supplementary Figure S1).

### Sensitivity of *peg-344* LAMP

**FIGURE 3:**
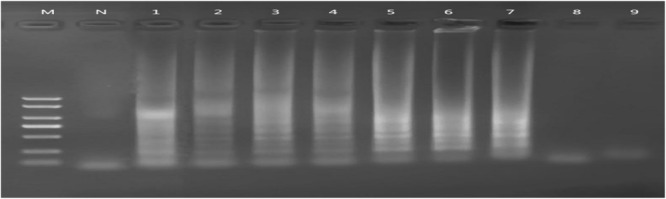
Sensitivity of *peg-344* LAMP. M: DL2000 DNA Marker; N: Negative control; 1∼9 DNA concentrations: 475, 47.5, 4.75, 4.75 × 10^– 1^, 4.75 × 10^– 2^, 4.75 × 10^– 3^, 4.75 × 10^– 4^, 4.75 × 10^– 5^, and 4.75 × 10^– 6^ng/μL, respectively.

We evaluated the sensitivity of the LAMP assay for detecting *peg-344*, serial dilutions of DNA were used. We got the genomic DNA of *K. pneumoniae* NTUH-K2044 whose concentration was about 475 ng/μL according to UV spectrophotometer. Then we diluted the DNA in a concentration gradient, with the original concentration, 10^–1^, 10^–2^, 10^–3^, 10^–4^, 10^–5^, 10^–6^, 10^–7^, and 10^–8^ dilutions used as templates for PCR and LAMP, respectively. Reaction results observed by electrophoresis on a 2% agarose gel. Comparing the sensitivity of LAMP and PCR, the minimum detection concentration of PCR was 47.5 pg/μL (not shown), while the minimum detection concentration of LAMP is 0.475 pg/μL (as shown in Figure 3). Thus, to detect *peg-344* from hvKp, LAMP is 100 times more sensitive than PCR.

### Direct Detection of *peg-344* by LAMP in Clinical Samples

**FIGURE 4:**
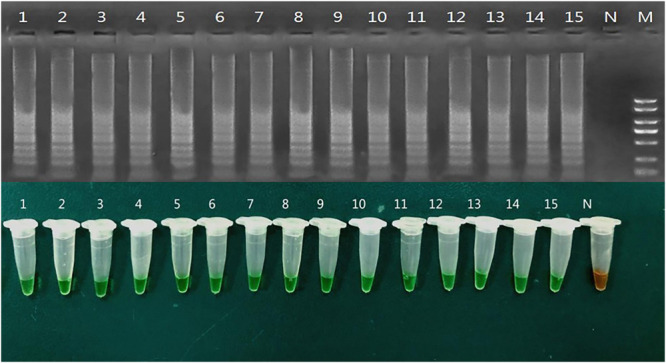
Direct detection of *peg-344* by LAMP in clinical samples. 1–15: as shown in Table 3 N: *Klebsiella pneumoniae* ATCC700603.

Twenty-eight bacterial isolates from the blood of patients infected with *K. pneumoniae* were used to detect the gene *peg-344* by LAMP as well as virulence assessment in Mouse lethality assay and Serum killing assay. As shown in Table 3 and Figure 4, the virulence phenotype of *K. pneumoniae* detected by LAMP was as precise as Mouse lethality assay. Mouse lethality assay revealed that 14 hypervirulent clinical strains had the 50% lethal dose (LD50) of less than 10^2^–10^3^ CFU, while 14 classical clinical strains had the LD50 of more than 10^6^ CFU. The Serum killing assay also was used to reveal the virulence phenotype of twenty-eight clinical *K. pneumoniae* strains and other hvKp marker genes (*iroB*, *iucA*, *rmpA*, *rmpA2*) were identified for the differentiation of hvKp from cKp confirmly (Supplementary Table S1).

**TABLE 3:** Direct detection of *peg-344* by LAMP in clinical samples.

**Number**	**Clincical strains**	**LAMP**	**LD50 (cfu)**	**Virulence phenotype^†^**
1	NTUH-K2044	+	9.5 × 10^1^	Hypervirulent
2	AY9293	+	1.8 × 10^2^	Hypervirulent
3	AY8972	+	5.4 × 10^2^	Hypervirulent
4	AY4992	+	3.6 × 10^2^	Hypervirulent
5	AY4970	+	4.8 × 10^2^	Hypervirulent
6	AY2075	+	8.2 × 10^2^	Hypervirulent
7	AY11489	+	8.9 × 10^1^	Hypervirulent
8	AP2841	+	1.2 × 10^2^	Hypervirulent
9	JDZK01	+	3.5 × 10^2^	Hypervirulent
10	GZK01	+	4.7 × 10^2^	Hypervirulent
11	GZK02	+	5.8 × 10^2^	Hypervirulent
12	GZK03	+	9.9 × 10^1^	Hypervirulent
13	GZK20	+	1.0 × 10^3^	Hypervirulent
14	AP855	+	1.9 × 10^2^	Hypervirulent
15	NUHL24835	+	2.5 × 10^2^	Hypervirulent
N	ATCC700603	−	>1 × 10^6^	Classical
17	GZ05	−	>1 × 10^6^	Classical
18	GZ04	−	>1 × 10^6^	Classical
19	JDZ02	−	>1 × 10^6^	Classical
20	AP1402	−	>1 × 10^6^	Classical
21	AY6324	−	>1 × 10^6^	Classical
22	XY18	−	>1 × 10^6^	Classical
23	XY1028	−	>1 × 10^6^	Classical
24	AY1109	−	>1 × 10^6^	Classical
25	AP34562	−	>1 × 10^6^	Classical
26	AY108	−	>1 × 10^6^	Classical
27	AY10513	−	>1 × 10^6^	Classical
28	AP1025	−	>1 × 10^6^	Classical
29	AP1201	−	>1 × 10^6^	Classical
30	AY2060	−	>1 × 10^6^	Classical

*^†^Interpretation of virulence was referred to reference (Siu et al., 2012). Hypervirulent strains with an LD50 of less than 1 × 10^3^ colony-forming units (CFU) are more likely to induce complications in mice. Classical strains with an LD50 of 1 × 10^6^ CFU of greater (do not cause complications).*

## Discussion

Compared to the classic *K. pneumoniae* (cKp) in hospital-acquired infection, the mortality of hvKp infection is higher because of frequently severe complications such as sepsis and subsequent metastatic spread (Choby et al., 2019). In particular, the emergence of carbapenem-resistant hvKp (CR-hvKp) has brought serious challenges to clinical treatment and basic research (Gu et al., 2018). Early and accurate diagnosis may reduce the morbidity and mortality caused by the hvKp infection, therefore, there is an urgent need for a rapid and sensitive diagnostic method.

The gene *peg-344* identified an isolate as a member of the hvKp-rich strain cohort with an accuracy of 0.97, a sensitivity of 0.99 and a specificity of 0.96. The second piece of evidence compared clinically defined hvKp-rich and cKp-rich strain cohorts in a murine sepsis model in which the gene *peg-344* was associated with a hazard ratio of severe disease or death > 25, and also supported his use to identify hvKp, according to a previous research (Russo et al., 2018). Most importantly, the gene *peg-344* appears to be hvKp-specific and, therefore has potential use as a rapid diagnostic test for the differentiation of hvKp from cKp (Bulger et al., 2017).

LAMP technology has been considered to be an excellent amplification and detection technology, with many advantages over traditional detection methods (Sheet et al., 2016). This assay is capable of specifically detecting *peg-344* in *K. pneumoniae* carrying virulence plasmids with high sensitivity within 60 min. The detection sensitivity of *peg-344* by LAMP assay is 100 times that of the corresponding PCR assay in this study. Several authors also have confirmed that LAMP has higher sensitivity and advantages compared to PCR reactions (Li et al., 2017). Moreover, the virulence phenotype of *K. pneumoniae* detected by LAMP was as precise as by Mouse lethality assay, and Serum killing assay in this study. Therefore, the LAMP assay is more suitable than a PCR assay for the rapid detection of *peg-344* in hvKp strains in clinical samples. Regrettably, the extremely few hvKp strains because of a deletion in the pLVPK-like virulence plasmid with gene *peg-344* lost maybe can’t be detected by *peg-*344 LAMP (Wyres et al., 2020).

The description of hvKp virulence genes remains incomplete, and it is still unclear which combination of genes are needed for maximal virulence (Russo and Marr, 2019). So the study has certain limitations, including the ambiguous definition of hvKp and the selection of biomarkers. When *peg-344* LAMP is suspected, PCR is the fastest way to detect these genes, and technologies such as real-time PCR and whole-genome sequencing will also be useful alternatives. Although these experiments are specific and exhibit high sensitivity, they rely on not only expensive equipment but also expertise to the analysis result. LAMP is a technology that can be performed easily even without using a thermal cycler. Its amplification time is much shorter than other molecular technologies. On the one hand, the LAMP does not lose its sensitivity and specificity, on the other hand, the LAMP is low in cost and easy to implement (i.e., RMB¥ 20 for LAMP vs. RMB¥ 25 for PCR). So LAMP can be an excellent epidemiological surveillance tool, especially in developing countries. However, the larger number of primers in LAMP may increase the primer-primer interactions. The product of LAMP is a series of concatemers of the target region, giving rise to a characteristic “ladder” or banding pattern on a gel, rather than a single band as with PCR. Furthermore, care should be taken when handling LAMP primers as opening the reaction tube could result in considerable contamination.

## Conclusion

A new, rapid, and simple assay for the detection of *peg-344* in hvKp was described in this study, which is amenable for point-of-care. Considering the proven ability to cause fatal diseases in healthy patients in the community, *peg-344* LAMP for hvKp will be a key tool in epidemiological investigations in underdeveloped areas.

## Data Availability Statement

The raw data supporting the conclusions of this article will be made available by the authors, without undue reservation, to any qualified researcher.

## Ethics Statement

The study has been evaluated by the Ethics Committee of the First Affiliated Hospital of Nanchang University.

## Author Contributions

QH and DL carried out the laboratory measurements. YL and LW made a significant contribution to the concept and design. DW and XL revised the manuscript for important intellectual content. WZ and YL took part in experimental design and data analysis. WL drafted the manuscript. All authors read and approved the final manuscript.

## Conflict of Interest

The authors declare that the research was conducted in the absence of any commercial or financial relationships that could be construed as a potential conflict of interest.

## Footnotes

1 https://primerexplorer.jp/lamp4.0.0/index.html

2 http://blast.ncbi.nlm.nih.gov/Blast.cgi
