# Supplementary material for: Rapid Detection to Differentiate Hypervirulent Klebsiella pneumoniae (hvKp) From Classical K. pneumoniae by Identifying peg-344 With Loop-Mediated Isothermal Amplication (LAMP)
Source: Front Microbiol. 2020 Jun 4;11:1189. doi: 10.3389/fmicb.2020.01189 (PMC7325879; doi:10.3389/fmicb.2020.01189)
Supplement: Supplementary file 1 [file Data_Sheet_1.doc]

A total of 28 *K. pneumoniae* strains isolated from the blood of patients were used for the *peg-344* standardization by PCR. The *K. pneumoniae* NTUH-K2044 and *K. pneumoniae* ATCC700603 were used as the positive control and negative control, respectively.


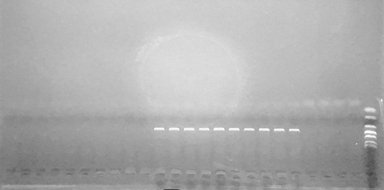


Figure S1 PCR analysis of a portion of clinical *K. pneumoniae* strains in this study
