# Supplementary material for: Rapid Detection to Differentiate Hypervirulent Klebsiella pneumoniae (hvKp) From Classical K. pneumoniae by Identifying peg-344 With Loop-Mediated Isothermal Amplication (LAMP)
Source: Front Microbiol. 2020 Jun 4;11:1189. doi: 10.3389/fmicb.2020.01189 (PMC7325879; doi:10.3389/fmicb.2020.01189)
Supplement: Supplementary file 2 [file Table_1.doc]

| **Isolates** | **Specimen**  **Table S1. Virulence phenotype of 28 Clinical *K. pneumoniae* isolates** | **Time of Strain collection** | **Ward** | **Patient outcome** | **Virulence genes (*iroB*, *iucA*, *rmpA*, *rmpA2*)** | **Serum killing assay** |
| --- | --- | --- | --- | --- | --- | --- |
| AY9293 | Blood | 2017/11/16 | ICU | Death | *iroB*, *iucA*, *rmpA*, *rmpA2* | Grade 6 |
| AY8972 | Blood | 2017/11/6 | Emergency ICU | Death | *iroB*, *iucA*, *rmpA*, *rmpA2* | Grade 5 |
| AY4992 | Blood | 2017/6/18 | Neurosurgery | Death | *iroB*, *iucA*, *rmpA*, *rmpA2* | Grade 5 |
| AY4970 | Blood | 2017/6/17 | Neurosurgery | Against Advise Discharge | *iroB*, *iucA*, *rmpA*, *rmpA2* | Grade 5 |
| AY2075 | Blood | 2017/2/22 | ICU | Death | *iroB*, *iucA*, *rmpA*, *rmpA2* | Grade 6 |
| AY11489 | Blood | 2018/1/25 | Gastroenterology | Discharge | *iroB*, *iucA*, *rmpA*, *rmpA2* | Grade 5 |
| AP2841 | Blood | 2017/2/8 | Emergency ICU | Death | *iroB*, *iucA*, *rmpA*, *rmpA2* | Grade 5 |
| JDZK01 | Blood | 2016/12/2 | Respiratory ICU | Death | *iroB*, *iucA*, *rmpA*, *rmpA2* | Grade 5 |
| GZK01 | Blood | 2016/4/20 | Neurosurgery | Against Advise Discharge | *iroB*, *iucA*, *rmpA*, *rmpA2* | Grade 5 |
| GZK02 | Blood | 2016/4/26 | Neurosurgery | Death | *iroB*, *iucA*, *rmpA*, *rmpA2* | Grade 5 |
| GZK03 | Blood | 2016/4/22 | Neurosurgery | Death | *iroB*, *iucA*, *rmpA*, *rmpA2* | Grade 5 |
| GZK20 | Blood | 2016/7/20 | Neurosurgery | Death | *iroB*, *iucA*, *rmpA*, *rmpA2* | Grade 6 |
| AP855 | Blood | 2018/4/10 | ICU | Death | *iroB*, *iucA*, *rmpA*, *rmpA2* | Grade 5 |
| NUHL24835 | Blood | 2016/2/22 | Gastroenterology | Death | *iroB*, *iucA*, *rmpA*, *rmpA2* | Grade 6  **Continued** |
| **Isolates** | **Specimen**  **Table S1. Continued** | **Time of Strain collection** | **Ward** | **Patient outcome** | **Virulence genes (*iroB*, *iucA*, *rmpA*, *rmpA2*)** | **Serum killing assay** |
| GZ05 | Blood | 2016/3/20 | Burn ICU | Discharge | - | Grade 1 |
| GZ04 | Blood | 2016/3/26 | Emergency ICU | Discharge | - | Grade 2 |
| JDZ02 | Blood | 2018/1/25 | Neurosurgery | Discharge | - | Grade 1 |
| AP1402 | Blood | 2017/2/16 | ICU | Death | - | Grade 2 |
| AY6324 | Blood | 2018/2/21 | Burn ICU | Discharge | - | Grade 1 |
| XY18 | Blood | 2016/5/20 | Emergency ICU | Discharge | - | Grade 2 |
| XY1028 | Blood | 2016/5/28 | Burn surgery | Discharge | - | Grade 1 |
| AY1109 | Blood | 2018/2/2 | ICU | Discharge | - | Grade 2 |
| AP34562 | Blood | 2017/3/19 | Neurosurgery | Discharge | - | Grade 1  Continued |
| AY108 | Blood | 2017/8/10 | Burn ICU | Discharge | - | Grade 2 |
| AY10513 | Blood | 2017/9/17 | Neurosurgery | Discharge | - | Grade 1 |
| AP1025 | Blood | 2018/1/21 | Emergency ICU | Discharge | - | Grade 2 |
| AP1201 | Blood | 2016/6/20 | Neurosurgery | Discharge | - | Grade 1 |
| AY2060 | Blood | 2017/5/18 | Burn surgery | Discharge | - | Grade 1 |
